# Virtual Hospital Induction for Medical Students: A Novel Approach

**DOI:** 10.7759/cureus.28244

**Published:** 2022-08-21

**Authors:** Vikramman Vignaraja, Joshua Creese, Staton Phillips, Anuhya Vusirikala

**Affiliations:** 1 Trauma and Orthopaedics, Basildon University Hospital, Essex, GBR; 2 Trauma and Orthopaedics, Anglia Ruskin University, School of Medicine, Essex, GBR

**Keywords:** medical education, medical student, digital, induction, virtual

## Abstract

Background and objective

Face-to-face hospital induction has been reported to lead to information overload with poor knowledge retention. The coronavirus disease 2019 (COVID-19) pandemic has provided an opportunity to redesign the induction process, thereby taking it into the digital age. In this study, we aimed to discuss a comprehensive and effective approach toward the induction of medical students.

Methods

A video was filmed on an iPhone (Apple Inc., Cupertino, CA), edited using the iMovie program (Apple Inc.), and shared with students before the start of the placement. It included a walk-through of the hospital and an explanation of educational opportunities. Pre- and post-placement questionnaires were distributed and focus groups were conducted.

Results

Our findings revealed that the participants strongly preferred virtual induction, feeling more confident about where to go, and who to contact, and better orientated on day one of their placement. They felt that being able to re-watch the induction at their convenience was invaluable.

Discussion

COVID-19 has brought about rapid digitalisation of medical services. The feedback from our study shows that virtual induction improved the well-being of students during their placement. By using easily accessible equipment, we have produced a useful resource that can be easily recreated by others.

## Introduction

Hospital placements for medical students are vital to help them acquire clinical skills, apply knowledge, and network for personal development. However, starting clinical placements in an unfamiliar hospital can be challenging. The General Medical Council (GMC) states that induction should be provided by all placements and should include familiarisation with physical settings and the layout of the placement environment so as to allow medical students to make the most of their hospital placement [1]. The induction programs for medical students by most hospitals consist of a whole day of didactic lectures, which can easily lead to information overload [2].

Coronavirus disease 2019 (COVID-19) was declared a global pandemic by the World Health Organisation on March 11, 2020, and the United Kingdom (UK) was placed under lockdown on March 23, 2020. Hospital footfall was reduced due to the increased risk of transmission. This led to a sudden halt in all medical student hospital placements [3]. As we slowly emerge from the pandemic, hospital placements are being reintroduced in a staged manner with a smaller number of students attending hospitals. The lack of in-person exposure to the clinical setting means that medical students are likely to feel apprehensive prior to starting their clinical placements.

Our pilot study aimed to provide a comprehensive and effective induction to medical students starting in our department during the pandemic to ease them back into the hospital environment. We created a virtual induction video that took them on a tour of the hospital, highlighting the areas of importance. This video was made available to them prior to starting placements. It was easily accessible on their mobile phones and computers, allowing them to refer to it as and when required to maximise their learning experience from day one instead of having to spend their whole first day of placement attending lectures.

## Materials and methods

Creating the virtual induction video

We developed a nine-minute virtual induction video for our hospital's trauma and orthopaedic department for medical students. Firstly, we created a working group that consisted of a trauma and orthopaedic consultant, registrar, core surgical trainees and medical students to obtain expertise from their various levels of knowledge and experience. We then created a hand-drawn storyboard that detailed each of the scenes in the video.

The information in the video provided orientation including a walk through the hospital highlighting the trauma meeting room, orthopaedic operating theatres, theatre changing rooms, wards, outpatient department, and plaster room. The video also included information on hospital services such as car parking, hospital ID badges, switchboard, and bleeping system. A link on “how to scrub” was also provided in the video for students to recap their scrubbing techniques. We filmed the video using an iPhone 11 Pro Max (Apple Inc., Cupertino, CA) and edited the video using the iMovie (Apple Inc.) editing software.

Study population

This was a prospective study involving mixed-method research undertaken in the trauma and orthopaedic department at a busy NHS acute general hospital in the UK. Eight second-year medical students started their hospital placement with the department - their first placement since the COVID-19 pandemic began in March 2020. The group of students were divided into pairs and each pair stayed in the department for one week over a four-week period in April 2021. Informed consent was obtained from all participants involved in the study. Ethical approval was deemed unnecessary as no patients were involved in the study, and this was confirmed with the education lead for medical students in the department.

Study design

Data was collected in the form of virtual focus groups and questionnaires. A focus group was conducted involving all participants to gather qualitative data prior to starting their clinical placements, followed by a questionnaire-based assessment to understand their previous hospital induction experience and current thoughts about starting clinic placements amid a pandemic (Appendix A). A further questionnaire-oriented analysis was conducted at the end of their placement to assess the usefulness of the virtual induction video (Appendix B). There was also a virtual focus group session at the end of the four weeks to debrief, assess the overall impact of the virtual induction video, and identify areas of improvement.

## Results

Pre-placement and post-placement focus groups were conducted and questionnaires were distributed to ascertain the impact of the virtual induction video. Among the eight participants, six (75%) had no prior placement experience at the hospital where this study took place. There were four male and four female students with an average age of 24 years.

Pre-placement analysis

All participants had face-to-face departmental induction during their placements, which had taken place before the COVID-19 pandemic. From this face-to-face induction, only 50% knew where to meet an allocated staff member on day one of their placement and over 90% did not know how to contact them whatsoever. Over 80% of students did not have confidence in and were dissatisfied with their ability to locate the departmental wards, clinics, and operating theatres after the information provided during their face-to-face induction. Six out of eight (75%) participants reported feeling either dissatisfied or very dissatisfied about the information they had received regarding extra-educational opportunities within the speciality where they had been placed pre-pandemic. The participants were then asked a set of questions relating to their upcoming trauma and orthopaedics placement. Participants’ knowledge of theatres in hospitals was limited. as none of the students knew where to find scrubs or how to scrub in for a case.

Post-placement analysis 

**Figure 1:**
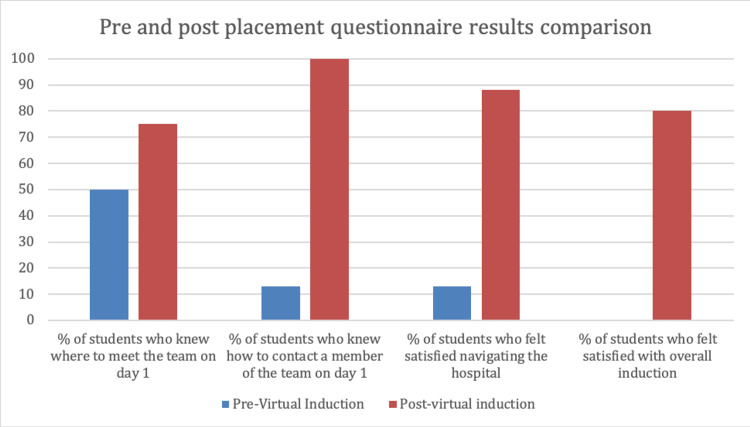
Graph showing results of student questionnaires taken before and after their orthopaedic placement Results from pre-virtual induction represent previous face-to-face inductions the students had. Post-virtual induction results represent the virtual induction used for the current placement

A post-placement questionnaire was distributed at the end of their one-week placement, the results of which, alongside the pre-placement questionnaire, are shown in Figure 1. Six (75%) out of eight participants reported that the information in the induction video helped with meeting up with the team on day one; this was an improvement from 50% of participants reporting the same from their previous face-to-face inductions. Participants responded positively when asked whether the video helped them to navigate around the hospital; 50% of participants agreed with this statement while 50% strongly agreed. This was a significant improvement from 80% of participants being dissatisfied with finding locations on their previous placements. Of note, 75% of participants reported that having access to the virtual induction video on their mobile phones and computers helped them feel more prepared in terms of who to contact/where to go if they got lost. This again was an improvement from the participants’ previous placements where 88% were unaware of how to contact staff.

Focus group results

Key comments from the two focus groups are presented in Table 1. We can see from the students' comments that they found traditional lecture-based induction unmemorable compared to virtual induction, which all students preferred, stating that this gave them greater autonomy over their learning and that being able to re-watch the induction video at their convenience was very useful.

**Table 1:** Key comments from pre- and post-placement focus groups

Pre- or post-placement	Responses
Pre-placement focus group	Several participants described their previous inductions as unmemorable and lecture-like
	2 participants in the focus group reported that much of their induction process had to be “figured out by themselves” previously
Post-placement focus group	All students reported the virtual induction video was their preferred method of induction over traditional face-to-face induction
	Participants found that the video format was easy to rewind and re-watch in parts to gather all the key information
	Students felt virtual induction gave them more “autonomy” over their placement
	3 participants (37.5%) from the group mentioned that they had attended theatres during their placement and that the video was helpful in locating the theatres and scrubs
	2 students attended the plaster room session during their placement and they both reported that the video helped them locate the plaster room facilities
	Many of the students highlighted that the video helped them find the location of the trauma meeting and orthopaedic wards. Participants agreed that virtual induction was a far more efficient use of their time than traditional face-to-face induction

Feedback on the virtual induction video

Feedback about how to improve the virtual induction video highlighted two main aspects: introduction to the orthopaedic team during the video so that students had a face and a name of someone they would be working with on their first day. Secondly, the students felt that issuing a one-sheet summary of key information for their placement would be helpful.

## Discussion

The COVID-19 pandemic has brought an unprecedented challenge to our healthcare services, forcing us into an era of rapid change. The digitalisation of services has forged onwards with changes being made at an unseen pace [4]. Primary care and outpatient clinic appointments have been made largely virtual with online video consultations, and patient uptake of remote services has risen sharply.

Amidst this upheaval, medical students have been subject to vast amounts of disruption such as the cancellation of clinical placements and severe restrictions on the patients they are allowed to interact with. It is known that medical students already suffer from greater levels of mental health disorders, stress, and burnout compared to the general public, and this has only been heightened by the uncertainty of the COVID-19 pandemic [5,6].

One silver lining of the pandemic is that it has given us the opportunity to digitally innovate. Our working group set about to improve the medical student experience as they were being introduced back into hospitals after a long period of hospital placement cancellations. Robust site and department-specific inductions have long been highlighted as crucial in welcoming newcomers, highlighting learning opportunities for students, and enabling a hassle-free transition into an alien environment [7]. To better support students returning to clinical placements, we decided to create a virtual induction video that would orientate students to their surroundings, highlight learning opportunities, and show who they could contact if they had any concerns as laid out by the GMC’s “clinical placement for medical students” [1].

Hospital induction can be broken down into two parts: corporate induction and departmental induction. Corporate induction covers statutory requirements of the trust, health and safety, and occupational health; whereas departmental induction covers orientation in the clinical workspace, introduction to the clinical team, and identification of learning opportunities [8].

As there is no existing published literature on virtual induction, our literature review focused on common problems reported by doctors regarding their usual face-to-face hospital induction. In 2020, the GMC commissioned an independent review into the nature of the problem with inductions and it was revealed that there are huge variations in the quality of induction [8]. Healthcare professionals (HCPs) felt that the focus was often placed on corporate inductions and was delivered for the benefit of the trust rather than the benefit of the HCPs. Those interviewed as part of the report felt that corporate induction should be simplified with more importance given to departmental induction [8]. It is the departmental induction that our working group has attempted to modernise given the department is where the majority of the learning during a hospital placement takes place, and it is where medical students gain an insight into that specialty.

Our pre-placement questionnaire was designed to gauge the effectiveness of previous face-to-face inductions. The questionnaire showed that despite face-to-face induction offering an opportunity to engage more with participants and answer any questions they may have, only 50% gained knowledge as to where they had to be on day one of their placement, and 90% did not know how to contact a named staff member. We hypothesised that in-person induction often overloads the participant with too much information, causing important information to be disregarded. The post-placement questionnaire found that 70% of participants felt that the virtual induction helped with meeting the team on the first day of placement - an improvement from face-to-face inductions. Time and time again we find junior doctors stating that face-to-face induction is “uninspiring” and a “tick-box exercise” [9]. The solution to this is multifaceted and we feel that by employing virtual induction, we can make sure new starters are introduced to their new environment in some form before they have even stepped onto hospital grounds.

Our study revealed that despite hospital maps being handed out at face-to-face induction and tours of the hospital, seven out of eight students (87.5%) were dissatisfied with their ability to navigate through the hospital. We wanted to focus on this and relate it to the GMC's “clinical placement for medical students”, which mandates that induction should provide “familiarisation with the physical setting and layout of the placement environment” [1]. We tackled this problem by providing an over-the-shoulder video walk-through of the hospital, focusing on the wards students would be placed on, operating theatres, fracture clinic, trauma meeting room, and canteen, while starting from the entrance of the hospital.

In our post-placement questionnaire 50% of students “agreed” that our virtual induction helped them navigate through the hospital, whereas the other 50% “strongly agreed”. Furthermore, in our post-placement focus group, one of the key benefits highlighted by students was that they could replay the virtual induction at their convenience. The concept of using videos in educational settings to allow independent learning with the ability to re-watch at a convenient time has been well evidenced [10] in other domains of medical education; however, this is the first example of the use of videos for departmental induction in the literature.

Clinical placements vary in their length. In shorter placements, spending even half a day for induction can lead to a significant reduction in time for potential learning. Pre-recorded virtual induction gives students an opportunity to receive induction before they even start their placement, saving valuable time that would be better spent on learning. In an age where students are shown to exhibit more consumer-like behaviour than ever before, catering to student needs and adding maximum value during these short placements are a must [11,12].

In our pre-placement focus group, students complained about the duration of face-to-face induction, which gave them a lot of unnecessary information that they found “unmemorable”. By condensing the information into a short video, induction is made more palatable, and the ability to re-watch was described by students as increasing their “autonomy”. Increasing autonomy in medical education has been shown to promote active engagement in an educational program and student well-being, leading to improved educational outcomes [13,14]. Providing induction in this way will help students in the long run, optimising their efficiency while on clinical placement.

From a departmental point of view, the major benefit of a virtual induction is that it does not require anyone to take time out of scheduled clinical time to induct new students. Especially with shorter placements, there is a rapid turnover of students, and this can add to a significant amount of clinical time that is given up for this purpose.

The authors recognize the limitations of this study. Firstly, the sample size was small; however, this was a pilot study testing how well this novel method of induction would be received prior to trialing it on the entire cohort. In order to counter the small sample size, the authors used focus groups to make the qualitative data more robust. Focus groups have long been used to improve the validity of qualitative data collected in questionnaire format, by highlighting any other significant issues participants may have and want to raise that are not measured in a questionnaire [15]. Secondly, as this was the first hospital placement after 18 months, students were attending in pairs for a short period of time. This meant that students could not capitalize on all learning opportunities that were shown to them in the virtual induction video, such as those relating to operating theatres and plaster rooms. Going forward, it will be important to trial this method of virtual departmental induction in larger groups of students.

## Conclusions

With our virtual induction video, we have created a useful tool for those starting in a new environment. We have shown that induction does not need to be lengthy nor cut into the valuable time at the beginning of the placement. Video induction enables more effective orientation, navigation, and learning, and improves the well-being of medical students during their placements. Our approach has shown that such a resource can be easily recreated by other departments for their individual needs with limited equipment, expenditure, and expertise.

## Figures and Tables

**Figure 2:**
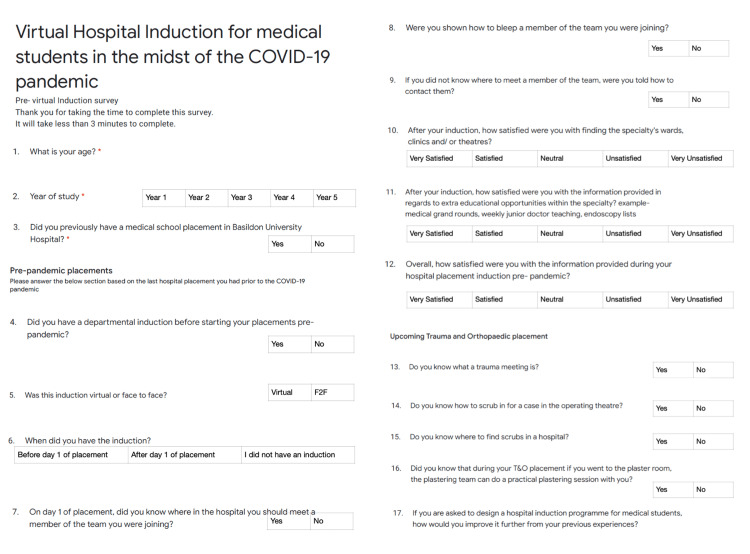
Appendix A - pre-placement questionnaire

**Figure 3:**
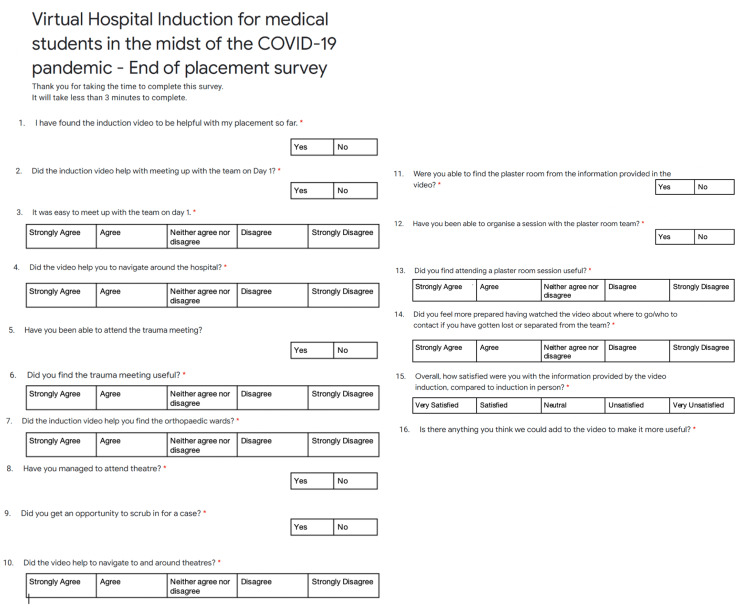
Appendix B - post-placement questionnaire
